# Progress in Surgery

**Published:** 1897-06-05

**Authors:** 


### PROGRESS IN SURGERY.

### SURGERY OF THE SPINE.

**The Surgical Treatment of Potts' Disease.**—The success attending surgical interference in carefully selected cases of Potts' disease is steadily increasing, and many new cases are now being reported. One of the most important factors in determining a successful issue to an operation on the spine is an accurate localisation of the vertebral lesion. Victor Horsley[1] points out that the tendency is, from an anatomical point of view, towards operating too low. He, therefore, indicates one or two points of practical importance in the localisation of spinal lesions. The cervical enlargement is easy of access, and is easily localised, the second and seventh cervical spines being readily recognised, as are also the intervening spines, with the exception of the third. In this region the cervical nerve roots and the corresponding spines are practically opposite one another.

In the dorsal region, it is useful to remember that the first dorsal nerve is the last large trunk of the brachial plexus. The second dorsal nerve contains the pupillary fibres, and also sends some fibres to the small muscles of the hand. Hence its importance. In this region the tip of a given spine is opposite a point in the cord from one to two and a half segments lower than might be expected. Thus there is danger of localising a lesion too low.

In the lumbar region, the origin of the third lumbar nerve roots is opposite the upper border of the laminæ of the twelfth D. vertebra, and the cord terminates one and a half vertebræ lower.

The cauda equina begins opposite the first lumbar vertebra as a rule. The exact localisation of function is also important, and Horsley considers this subject under the following heads:—

(1) *Movement.*—The cervical enlargement is clearly divided into various zones representing very definite groups of muscles. Thus the fifth and sixth roots supply the deltoid, biceps, and supinator longus (Erb's group). The eighth cervical and first dorsal supply the flexors of the fingers and small muscles of the hand.

(2) *Sensation.*—The upper extremity may be divided into a radial side and an ulnar side by a line drawn up the middle (the arm being in the anatomical position). The radial side is supplied by the posterior roots of the fifth, sixth, and seventh cervical nerves, and the ulnar by the seventh and eighth cervical and first dorsal nerves. This applies to tactile and temperature sensation and perhaps to muscular sense. Pain is conducted by the central portions of the cord.

(3) *Representation of Viscera.*—The heart and lungs get fibres from the upper dorsal nerves; the abdominal viscera (chiefly for their blood vessels) from the lower dorso-lumbar region. The cardio-accelerator fibres come down the lateral column and leave by the eighth C. and first D. roots.

The pelvic viscera (bladder and rectum) being supplied by the long nerve-roots of the cauda equina, may be disturbed by any lesion involving these nerves from the cord down to their termination. Hence symptoms referable to these organs are of limited value for purposes of localisation.

All the abdominal organs are represented by that portion of the cord between the upper border of the twelfth D. and the middle of the second L. vertebra, all of which may be exposed by removal of the arches of the twelfth D. and first L. vertebræ.

(4) *The Pupillary Fibres* leave by the second D. nerves, and may be of great value in diagnosis.

(5) *Heat Regulatory Fibres.*—Horsley believes that the heat-regulating centres are in the cortex of the Rolandic area and that the damage of fibres from these centres in any part of their course down to the periphery will be liable to produce a rise of temperature. These fibres being more numerous in the upper part of the cord, lesions there will produce a correspondingly greater thermic disturbance.

The writer also discusses the question of caries, or suppurative tuberculous disease of the bodies of the vertebræ, which he says produces secondary paraplegia by simple compression of the cord. There is marked perithecal inflammation, but the cord itself does not share in the tuberculous disease. Myelitis is set up by occlusion of its vessels due to pachymeningitis, non-tuberculous in nature. Cases above 30 years of age are much less satisfactory for surgical interference. Severe pain is a rare symptom of dorsal caries. It is important to determine the presence or absence of myelitis before undertaking an operation. In adults it contra-indicates operation entirely, but not so in children, who often recover from it. The presence of analgesia is a valuable sign in the diagnosis.

In the general treatment extension in bed, for six months or longer, is recommended in preference to Sayres' jacket. If an abscess forms surgical intervention is called for at once. Rise of temperature, spasticity, and great exaggeration of the deep reflexes are indicative of abscess formation. The operation consists in removing the laminæ, removal of all perithecal thickening, evacuation of pus, scraping carefully all diseased tissue away, and removing sequestra, if necessary gaining access by division of pedicles and transverse processes. Recovery is often remarkably rapid.

Wachenhusen[2] believes that the operation of laminectomy is justifiable and rational, even in cases where paralysis has lasted for some time, because this is due to a disturbance of circulation, which may last for a long time without irreparable changes taking place in the cord. He thinks that operation is always indicated when the caries is in the vertebral arch. When the body of the bone is affected the operation should be done if the symptoms have not lasted so long as to have suggested irreparable damage to the cord, and when prolonged orthopædic treatment has failed to produce benefit.

Owen[3] doubts the value of laminectomy in paraplegia from caries; but Parkin strongly advocates it. A successful operation for compression associated with Potts' disease is reported by Punton, of Kansas.[4] The points of special interest about the case are: (1) The age of the patient; he was 27. (2) The entire absence of evidence of personal or inherited predisposition to tuberculosis. (3) There was no cachexia or emaciation. (4) There was no evidence of trauma, exposure, syphilis, or other common cause for spinal caries. (5) There was no elevation of temperature throughout the course of its development. (6) There was no pain, even on pressure or movement, at the seat of the lesion. (7) No atrophy, bed sores, or other trophic disturbances. (8) The positive evidences of the disease were neuralgic bodily pains; a swelling about the size of a hickory nut over the sixth dorsal spine, which gradually increased for about six months, gradually increasing weakness of the lower limbs, ending in complete paralysis, and marked angular curvature. (9) Laminectomy of the fifth and sixth dorsal vertebræ was at once followed by improvement. In about ten days the patient gained the entire use of his limbs, and the paralysis, which had lasted for about eight months, disappeared entirely. Four weeks later, however, symptoms reappeared, and a second operation was required to evacuate a large abscess. After this complete recovery took place.

Parona[4] records two cases of caries in adults, in which local recovery followed operation from behind. In neither case was there deformity of the spine.

Anderson,[5] in a boy of nine, who had suffered from paraplegia for 18 months, obtained marked improvement within a week of performing laminectomy, all signs of spinal irritation having passed off. The writer made a mesial longitudinal incision over the diseased vertebræ, and he points out the importance of almost completely dividing the laminæ with the saw before using the bone pliers to avoid injuring the dura mater.

Another very satisfactory result is reported by Kirk,[6] of Belfast, in the case of a girl, aged five and a half years, who suffered from loss of power in the lower limbs, with rigidity, increased patellar reflex and ankle clonus, and want of control of bladder and rectum. There was complete analgesia and almost complete anæsthesia as high up as the lower part of the thorax. There was almost no deformity.

Menard's operation was performed. A longitudinal incision, four and a half inches long, was made just internal to the tips of the transverse processes of the fourth and fifth dorsal vertebræ. The transverse processes were cut through at their bases, freed from the ribs and removed. The ribs were cut through internal to their angles, and the heads and necks removed. In this way access was obtained to the diseased area, and *débris* representing the body of the fifth and part of the fourth dorsal vertebræ was removed. The pleura was punctured, but no evil resulted. Recovery soon set in, and some months after the patient was able to walk with a little assistance.

**Sacro-iliac Disease.**—In reporting a case of sacro-iliac disease, Judge-Baldwin[7] gives a short *résumé* of the literature of the subject, with some remarks on its symptomatology and treatment. The disease may be met with in one of two stages—suppurative, which is the commoner and the more serious (7·9 per cent. only recovering); or non-suppurative, where 94 per cent. recover. The leading symptom is pain, gnawing or rheumatic in character, extending to the anus, and sometimes to the knee; aggravated by movement and by pressing the ilia together. There is also tenderness over the joint, and the patient cannot lie on the affected side. Lameness, with short step and apparent shortening of the leg; swelling over the joint, with atrophy of gluteal muscles; and evidences of pus formation are also present. The writer's case was that of a married woman, aged twenty-six, who began to suffer from symptoms of sacro-iliac disease five and a half months after the birth of her first child. An abscess formed in the front of the left thigh. This was opened, and found to contain about a pint of thin pus. The sacro-iliac joint was then explored, and all diseased tissue removed, and a drainage tube passed through from one wound to the other. The wounds slowly closed, and eventually the patient made a complete recovery.

[1] Clinical Journal, Jan. 13, 1897. [2] Beiträge z. Klin. Chirurg., Bd. XVII. [3] Jour. of Nervous and Mental Diseases, Dec., 1896. [4] Brit. Med. Jour., June, 1896. [5] Med. Press and Circular, June, 1896. [6] Brit. Med. Jour., Nov., 1896. [7] Brit. Med. Jour., Dec. 1896.

